# Metabolic profile of mesenchymal stromal cells and macrophages in the presence of polyethylene particles in a 3D model

**DOI:** 10.1186/s13287-023-03260-4

**Published:** 2023-04-21

**Authors:** Victoria Teissier, Qi Gao, Huaishuang Shen, Jiannan Li, Xueping Li, Elijah Ejun Huang, Junichi Kushioka, Masakazu Toya, Masanori Tsubosaka, Hirohito Hirata, Hossein Vahid Alizadeh, Chima V. Maduka, Christopher H. Contag, Yunzhi Peter Yang, Ning Zhang, Stuart B. Goodman

**Affiliations:** 1 Department of Orthopaedic Surgery, Stanford University School of Medicine, Stanford, CA USA; 2 Department of Material Science and Engineering, Stanford University School of Medicine, Stanford, CA USA; 3 Institute for Quantitative Health Science and Engineering, Departments of Biomedical Engineering and Microbiology and Molecular Genetics, Michigan State University, Michigan, USA; 4 Department of Bioengineering, Stanford University, Stanford, CA USA; 5 Biomedical Innovations Building, Orthopaedic Research Laboratories 0200, 240 Pasteur Drive, Palo Alto, CA 94304 USA; 6 Redwood City, USA

**Keywords:** MSCs, Macrophage, Polyethylene particles, Mitochondria, Metabolism, Three-dimensional model

## Abstract

**Background:**

Continuous cross talk between MSCs and macrophages is integral to acute and chronic inflammation resulting from contaminated polyethylene particles (cPE); however, the effect of this inflammatory microenvironment on mitochondrial metabolism has not been fully elucidated. We hypothesized that (a) exposure to cPE leads to impaired mitochondrial metabolism and glycolytic reprogramming and (b) macrophages play a key role in this pathway.

**Methods:**

We cultured MSCs with/without uncommitted M0 macrophages, with/without cPE in 3-dimensional gelatin methacrylate (3D GelMA) constructs/scaffolds. We evaluated mitochondrial function (membrane potential and reactive oxygen species—ROS production), metabolic pathways for adenosine triphosphate (ATP) production (glycolysis or oxidative phosphorylation) and response to stress mechanisms. We also studied macrophage polarization toward the pro-inflammatory M1 or the anti-inflammatory M2 phenotype and the osteogenic differentiation of MSCs.

**Results:**

Exposure to cPE impaired mitochondrial metabolism of MSCs; addition of M0 macrophages restored healthy mitochondrial function. Macrophages exposed to cPE-induced glycolytic reprogramming, but also initiated a response to this stress to restore mitochondrial biogenesis and homeostatic oxidative phosphorylation. Uncommitted M0 macrophages in coculture with MSC polarized to both M1 and M2 phenotypes. Osteogenesis was comparable among groups after 21 days.

**Conclusion:**

This work confirmed that cPE exposure triggers impaired mitochondrial metabolism and glycolytic reprogramming in a 3D coculture model of MSCs and macrophages and demonstrated that macrophages cocultured with MSCs undergo metabolic changes to maintain energy production and restore homeostatic metabolism.

## Introduction

Mesenchymal stromal cells (MSCs) are multipotent cells that can differentiate into multiple phenotypes: osteogenic, chondrogenic, adipogenic, connective tissue stroma and others [1]. MSCs stimulate bone formation and repair and have immunomodulatory capacities on other cell types, including macrophages [2]. Macrophages are immune cells specialized in detection, phagocytosis and destruction of harmful stimuli. Contaminated polyethylene particles (cPE), first studied in the field of periprosthetic osteolysis, have become a validated model for chronic inflammatory bone destruction [3–6]. During this process, macrophages mediate a biological cascade that (a) phagocytoses cPE, (b) stimulates the differentiation of uncommitted naïve M0 macrophages into pro-inflammatory M1 or anti-inflammatory M2 macrophages, (c) induces both osteoclastic and osteoprogenitor cell differentiation, (d) stimulates angiogenesis and (e) releases cytokines in the microenvironment [7–10]. The cross talk between MSCs and macrophages is a two-way channel of communication [11, 12]: MSCs modulate macrophage polarization state and macrophages promote MSC differentiation, migration or apoptosis.

Mitochondria are intracellular organelles that regulate cellular homeostasis, energy production, generation of reactive oxygen species (ROS) and control of apoptosis [13]. Two main pathways lead to ATP production. While glycolysis relies on anaerobic cytoplasmic reactions converting glucose to pyruvate and generating ATP, oxidative phosphorylation is the metabolic pathway of aerobic mitochondrial reactions using electrons produced by the tricarboxylic cycle (TCA) to produce ATP. Warburg discovered that cancer cells preferentially use the glycolytic pathway, even under normal oxygen concentration [14]; the Warburg effect—also called aerobic glycolysis—has since been described in non-cancerous cells [15].

Mitochondria also coordinate adaptation to the local microenvironment. While uncommitted MSC metabolism relies on glycolysis, differentiated cells mostly use oxidative phosphorylation, and this bioenergetic switch—although unexplained—is crucial to MSC differentiation [16]. Several mitochondrial pathways modulate the direction of MSC differentiation: Excess ROS impairs osteogenic commitment, and PGC-1α (a regulator of mitochondrial biogenesis) enhances adipogenesis [17]. Mitochondria modulate macrophage polarization: M1 macrophages mostly use glycolysis, while M2 macrophages rely on oxidative phosphorylation for ATP production [18, 19]. Little is known about the role of mitochondrial function during cPE-induced inflammation or how the MSC-macrophage interactions affect mitochondrial metabolism [11, 15, 19].

We hypothesized that (a) exposure to cPE leads to impaired mitochondrial metabolism and glycolytic reprogramming and (b) macrophages play a key role in driving the inflammatory response to cPE and affecting MSC function. To test this hypothesis, we established a 3D model in which we cultured MSCs with or without naïve M0 macrophages and with or without cPE in Gelatin Methacrylate (GelMA) to evaluate the effects of MSC-macrophage cross talk on mitochondrial metabolism, energetic pathways, macrophage polarization and MSC osteogenic differentiation.

## Material and methods

### Cell culture and scaffold

The methods of isolating mouse bone marrow-derived MSCs and macrophages have been described previously [20]. To harvest macrophages and MSCs, bone marrow cells were collected from 10 C57BL/6 J female mice aged 8–10 weeks old obtained from Jackson Laboratory (Bar Harbor, Maine, USA). Institutional guidelines for the care and use of laboratory animals were observed in all aspects of this project. Mice were housed in a specific pathogen-free facility with a 12-h light, 12-h dark cycle and given free access to food and water. Animals were euthanized by carbon dioxide (CO_2_) inhalation. Under sterile conditions, the femora and tibiae of the mice were surgically removed. Using a 25-gauge needle, the bone marrow was flushed into a 50 mL centrifuge tube by injecting 5 mL of macrophage basal medium (RPMI Medium 1640 supplemented with 10% fetal bovine serum, 1% antibiotic–antimycotic (Invitrogen, Grand Island, NY)) or 5 mL of MSC basal medium (MEM alpha, supplemented with 10% FBS, 1% antibiotic–antimycotic (Thermo Fisher Scientific, Waltham, MA, USA). After filtration (70 µm), cells were spun down (400 g, 10 min) and resuspended in 1 mL/tube ice cold red blood cell lysis buffer (Invitrogen) for 2 min (min) at 4 °C, followed by the addition of 20 mL/tube basal medium.

The naïve macrophages (M0) were cultured in macrophage differentiation medium (macrophage basal medium supplemented with L929 Cells-Conditioned Medium (LCM) and 100 ng/mL M-CSF (R&D Systems, Minneapolis, MN, USA)) for 48 h (h). MSCs were cultured in MSC basal medium and passaged until P8. The isolation protocol was previously validated by characterizing the immunophenotype of isolated MSCs at passage 4: spinocerebellar ataxia type 1 (Sca1 +)/CD105 + /CD44 + /CD34-/CD45-/CD11b- [20].

The GelMA was synthesized according to a previously described protocol [21]. Briefly, gelatin was dissolved in deionized water (DI) (10% weight per volume) at 50 °C. Methacrylic anhydride was added to gelatin solution at a molar ratio of 100:1, and the solution was allowed to react under stirring for 1 h at 50 °C. The mixture was then diluted 5 times with DI water and dialyzed against DI water using a dialysis tube (Spectrum Laboratories, Rancho Dominquez, CA) with 6 − 8 kDa molecular weight cutoff for 3 days at 40 °C. The obtained solution was freeze-dried and stored at − 80 °C. The degree of substitution of resulted GelMA was 73.2%.

MSCs were cocultured with or without 0.125% polyethylene particles (PE) coated with 10 ng/mL lipopolysaccharides (LPS) (hereafter referred to as cPE) and with or without macrophages in a 1:1 ratio in the 3D hydrogel scaffold. Briefly, MSCs and macrophages were suspended in a 15% GelMA solution (pH 7) at a concentration of 10 M/mL, and the suspension was photopolymerized in 50 µl molds under UV light (3.4 mW/cm^2^, 90 s, OmniCure S2000, Excelitas Technologies, Canada). The constructs were cultured with 50% osteogenic medium (MSC basal medium supplemented with 10 nmol/L dexamethasone, 10 mmol/L β-glycerol phosphate and 50 μmol/L ascorbate-2-phosphate) and 50% macrophage differentiation medium. Nine constructs per group were harvested for analysis at 2 timepoints, day 7 and day 21.

### Live cells mitochondrial staining

For each group, n = 2 scaffolds were used for live cell mitochondrial staining. Each scaffold was separated into 2 halves for two independent staining probes.

First, mitochondrial membrane potential was assessed by tetramethylrhodamine, methyl ester (TMRM) staining: Half of the scaffolds were incubated with 1 mL of 250 nM TMRM (Invitrogen™, T668) in complete medium (50% osteogenic medium 50% macrophage stimulating medium) for 30 min at 37 ℃ and washed three times with PBS before images acquisition.

Second, mitochondrial superoxide and ROS were stained with mtSOX Deep Red (Dojindo, MT14-10): The other half of the scaffolds were incubated with 1 mL of 10 μmol/L mtSOX Deep Red solution in HBSS for 30 min at 37 ℃ and washed twice with HBSS before image acquisition. mtSOX Deep Red is selectively oxidized by superoxide and ROS in the mitochondria; thus, signal increases with superoxide/ROS production.

Nuclear counterstaining was performed with 300 nM DAPI (4′,6-Diamidino-2-Phenylindole, Dihydrochloride).

Fluorescence intensity was read immediately after staining using a Leica DMI8 confocal microscope with Leica STELLARIS 5 Scan Head confocal microscope with 200 × magnification with an excitation wavelength of 540 nm and an emission wavelength of 590 nm for TMRM, Ex: 550 nm, Em: 675 nm for mtSOX and ex: 358 nm, Em: 461 nm for DAPI. Fluorescence intensity in all scaffolds was measured in three randomly selected areas in a 100-µm-thick z-stack area. Two independent experiments were performed.

Mean fluorescence intensity, median, minimum, maximum were measured with Fiji (Fiji Is Just ImageJ) software (http://fiji.sc/, plugins 3D ImageJ Suite and Image Science).

### Flow cytometry

For each group, n = 3 scaffolds were used. Single-cell suspensions of scaffolds were prepared by a combination of enzymatic digestion (2 mg/mL Collagenase) and mechanical disruption (platform shaker at 37 °C for 60–90 min, Medimachine System, BD Biosciences). Cells were harvested after centrifugation at 400 g for 5 min and passed through 70 µm and 40 µm cell strainer.

Briefly, beads were prepared and after adding Fc block, cells were stained for 30 min at 4 °C in dark with fluorescein isothiocyanate (FITC)-conjugated anti-CD11b (diluted 1:200, Thermo Fisher Scientific); peridinin–chlorophyll–protein complex (PerCP)-eFluor710-conjugated anti-CD80 (diluted 1:200, Thermo Fisher Scientific); APC-Cy7-conjugated anti-CD206 (diluted 1:300, Thermo Fisher Scientific); and Alexa700-conjugated anti-CD44 (diluted 1:50, Bio-Rad Laboratories). Cells were washed with PBS after antibody staining and mitochondrial staining was performed with allophycocyanin (APC)-conjugated mtSOX Deep Red (10 μmol/l, Dojindo) for 30 min at 37 °C. Single stained beads for each antibody were prepared for compensation. Cells were washed 2 times with PBS, resuspended in 300–500 µL of PBS with the density of 1–10 M/mL, and dead cells were stained by adding to each sample 2.5 µl of propidium iodide (PI) (diluted 1:300, Thermo Fisher Scientific) just prior to analysis.

Cells were analyzed using a BD LSR II Flow Cytometer (BD) equipped with FACSDiva Version 6.1.1 software (BD Biosciences). FACS data were further analyzed using FlowJo™ v10.8 Software (BD Life Sciences).

### qPCR analysis

RNA was extracted from n = 3 scaffolds in each group by TRIzol reagent and miRNeasy kit (Qiagen, mat. No. 1071023). Purity was measured with NanoDrop One (Thermo Fisher Scientific, Waltham, MA, USA). RNA was reverse transcribed into cDNA by the High-Capacity cDNA Reverse Transcription Kit (Applied Biosystems, 4,368,814).

Gene primers were used for quantifying the expression of *Fn1* as an MSC marker, *Runx2, Collα1, Opn, Ocn* for MSC differentiative capacity*, Pfkfb3, Pkm2, Hif1α* for mitochondrial glycolytic reprogramming*, Tnfα, Il-1β, Nos2, Il-6, Arg1* for inflammatory reaction and *Pgc-1α, Errα, Creb, Nrf1* for response to stress and mitochondrial metabolism evaluation (Table 1) with PowerTrack SYBR Green Master Mix protocol (Thermo Fisher Scientific). Analysis was performed on day 7 and day 21.

**Table 1:** Primers sequences

Primer name	Sequence (5'-3')	
TNFa	*Forward*	TCTCATGCACCACCATCAAGGACT
	*Reverse*	ACCACTCTCCCTTTGCAGAACTCA
IL-1b	*Forward*	AAGGGCTGCTTCCAAACCTTTGAC
	*Reverse*	ATACTGCCTGCCTGAAGCTCTTGT
IL-6	*Forward*	ATCCAGTTGCCTTCTTGGGACTGA
	*Reverse*	TAAGCCTCCGACTTGTGAAGTGGT
Nos2	*Forward*	TCTTTGACGCTCGGAACTGTAGCA
	*Reverse*	ACCTGATGTTGCCATTGTTGGTGG
Arg1	*Forward*	CTGGAACCCAGAGAGAGCAT
	*Reverse*	CTCCTCGAGGCTGTCCTTT
RUNX2	*Forward*	CTACCCAGCCACCTTTACCTAC
	*Reverse*	GAACTGATAGGATGCTGACGAAG
Col1a1	*Forward*	GTGGTGACAAGGGTGAGACA
	*Reverse*	GAGAACCAGGAGAACCAGGA
SPP1/OPN	*Forward*	GACAACAACGGAAAGGGCAG
	*Reverse*	GATCGGCACTCTCCTGGCT
BGLAP/OCN	*Forward*	AGGAGGGCAATAAGGTAGTGAAC
	*Reverse*	AGGCGGTCTTCAAGCCATAC
FN1	*Forward*	TGGTGGCCACTAAATACGAA
	*Reverse*	GGAGGGCTAACATTCTCCAG
PGC-1a	*Forward*	AAACTTGCTAGCGGTCCTCA
	*Reverse*	TGGCTGGTGCCAGTAAGAG
NRF1	*Forward*	GCACCTTTGGAGAATGTGGT
	*Reverse*	GGGTCATTTTGTCCACAGAGA
ERRa	*Forward*	TACATCAAGGCAGAGGCAGC
	*Reverse*	CACACCGTAGTGGTAGCCAG
CREB1	*Forward*	TCAAGGAGGCCTTCCTACAG
	*Reverse*	GGGGCTGAAGTCTCCTCTTC
PFKFB3	*Forward*	AGAACTTCCACTCTCCCACCC
	*Reverse*	AGGGTAGTGCCCATTGTTGAA
PKM2	*Forward*	TCGCATGCAGVACCTGATT
	*Reverse*	CCTCGAATAGCTGCAAGTGGTA
HIF-1a	*Forward*	ACCTTCATCGGAAACTCC
	*Reverse*	CTGTTAGGCTGGGAAAAG
Actin	*Forward*	CGGTTCCGATGCCCTGAGGCTCTT
	*Reverse*	CGTCACACTTCATGATGGAATTGA

### Enzyme-linked immunosorbent assay (ELISA)

The supernatants were collected at different timepoints (day 3, 7, 10, 14 and 21), centrifuged at 1,500 rpm for 10 min at 4 °C to remove cell debris and kept frozen until use. The secretion levels of TNFα, IL-4 and osteoprotegerin/TNFRSF11B by MSCs and macrophages following their exposure to contaminated particles were measured by enzyme-linked immunosorbent (ELISA) assays (Mouse Duoset ELISA DY410-05, DY404-05, DY459; RnD Systems) according to the manufacturer’s instructions. Absorbance was determined at 450 nm and 570 nm with wavelength correction. Osteoprotegerin/TNFRSF11B levels were calculated following a 1:5 dilution of the samples. Cytokine concentrations were calculated from the calibration curves obtained from serial dilutions of standard protein.

#### Histologic and immunohistochemical analysis

For each group, n = 1 scaffolds were fixed with paraformaldehyde (PFA) 4% overnight at 4 ℃ and then placed in 30% sucrose (Thermo Fisher Scientific, Waltham, MA, USA) at 4 °C After being embedded in the OCT compound (Thermo Fisher Scientific, Waltham, MA, USA) and frozen in − 80 °C, and the scaffolds were mounted onto a microtome and sectioned into 10-μm-thick slices. Sections were stained with hematoxylin (Vector Laboratories, Burlingame, CA) and eosin-Y solution (Sigma-Aldrich, St. Louis, MO, USA). Separate serial sections were also stained with 40 mM Alizarin Red S (Thermo Fisher Scientific, Waltham, MA, USA) and alkaline phosphatase (1-StepTM NBT/BCIP Substrate Solution) (Abcam, Cambridge, UK).

The ALP-positive area and ARS-positive area based on the entire area of the scaffolds were calculated using the image analysis software program QuPath version 0.3.2 [22].

For immunohistochemical analysis, specimens were blocked and permeabilized by 5% BSA with 0.3% Triton X-100 buffer for 60 min at room temperature, followed by primary and secondary antibody incubation. Macrophages were identified with rat anti-CD11b antibody (Abcam, Cambridge, MA, USA) followed by Alexa Fluor® 647 conjugated donkey anti-rat IgG (Abcam, Cambridge, MA, USA). M1 pro-inflammatory macrophages were stained by mouse anti-inducible nitric oxide synthase (iNOS) antibody (Abcam, Cambridge, MA, USA) followed by Alexa Fluor® 488 conjugated goat anti-mouse IgG (Invitrogen, CA, USA). M2 anti-inflammatory macrophages were identified with rabbit anti-liver arginase (Arg1) antibody (Abcam, Cambridge, MA, USA) followed by Alexa Fluor® 555 conjugated donkey anti-rabbit IgG (Invitrogen, Carlsbad, CA, USA). Slides were mounted by prolong gold antifade mount with DAPI (Life Technologies, Grand Island, NY, USA). Slides were imaged using a fluorescence microscope with 200 × magnification (BZ-X800, Keyence, IL, USA). Positive cells in all slides were counted in three randomly selected areas by two independent observers*.*

### Statistical analysis

The statistical analysis was conducted using Prism 8 (GraphPad Software, San Diego, CA). Data were expressed as mean with standard deviation. As indicated on figure legends, one-way ANOVA followed by Tukey post hoc test, Brown–Forsythe and Welch ANOVA followed by Games–Howell’s multiple comparison test or two-way ANOVA followed by Tukey post hoc test were performed to analyze the effects in the study. *P* < 0.05 was regarded as statistically significant.

## Results

### Macrophages restored the impaired mitochondrial function of MSCs exposed to cPE

The mitochondrial membrane potential—defined as the difference in electrical potential between the inner and outer mitochondrial membranes—is generated and maintained by the proton pump of the respiratory chain. Mitochondrial membrane potential is also related to the MSCs’ differentiative capacities and is increased during osteogenic differentiation [23]. When membrane potential drops because of altered or dysfunctional mitochondria, TMRM no longer accumulates, and signal is decreased. The role of reactive oxygen species (ROS) in inflammatory disorders and cancer is well established [24, 25]. When mitochondria are altered or under stress—such as inflammatory disorders—ROS production is increased and the mtSOX Deep red signal intensity increases.

**Fig. 1:**
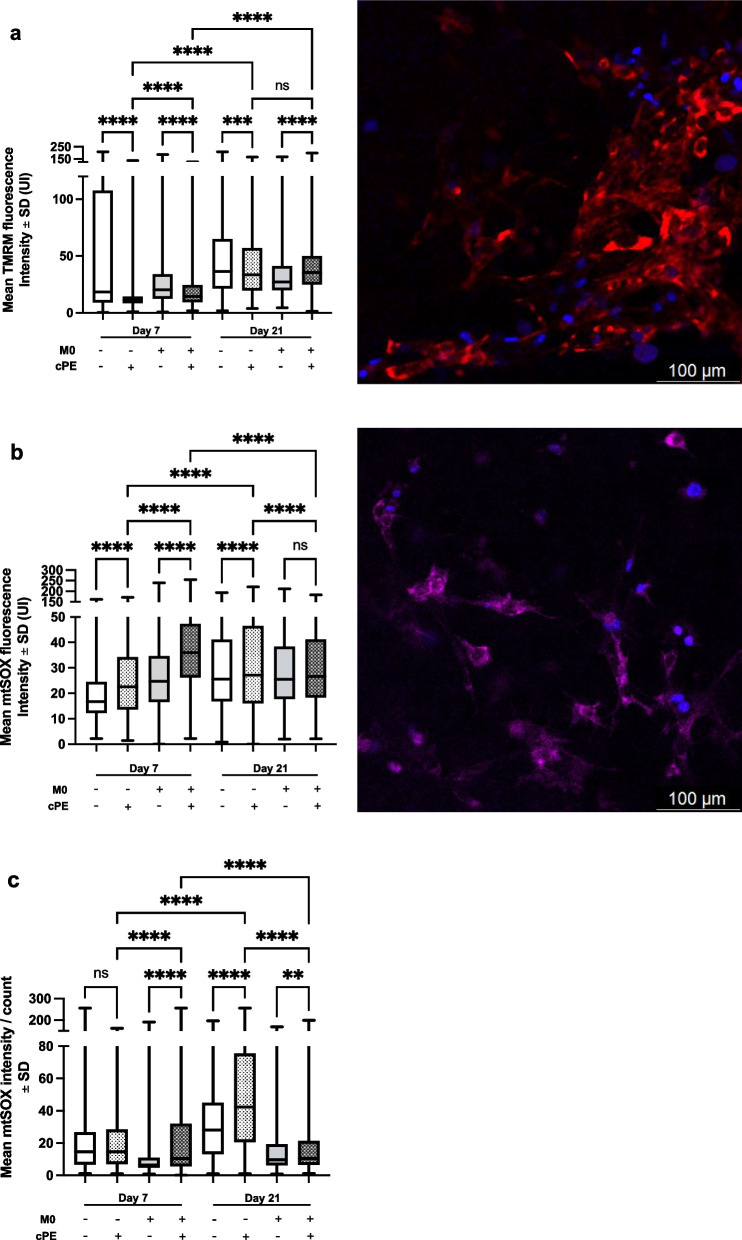
Mitochondrial function of MSCs: membrane potential and ROS production. **a**: TMRM staining followed by live cell confocal microscopy. **b**: mtSOX staining followed by live cell confocal microscopy. **c**: mtSOX staining followed by flow cytometry analysis. Box: mean and IC95%, min and max. *p < 0.05; ****p < 0.0001. One-way ANOVA followed by post hoc Turkey test between MSC and MSC + cPE; MSC + M0 and MSC + M0 + cPE; and MSC + cPE and MSC + M0 + cPE. ROS: reactive oxygen species, TMRM: Tetramethylrhodamine, methyl ester, M0: naïve macrophages, cPE: contaminated polyethylene particles

With the two samples available, we observed that TMRM signal was decreased and mtSOX signal was increased in the MSC + cPE group when compared to the MSC group, on day 7 and 21 *(P* < 0.0001; Fig. 1a and 1b). In the MSC group, exposure to cPE led to decreased membrane potential and increased mitochondrial ROS production. In other words, mitochondrial metabolism and integrity of MSCs were impaired with exposure to cPE. In the MSC + M0 + cPE group, when compared to MSC + M0, on day 7, TMRM signal was decreased (*P* < 0.0001) and mtSOX signal was increased (*P* < 0.0001); but at day 21, TMRM signal was restored (*P* < 0.0001), and ROS production was decreased (ns). We added macrophages in the culture system to evaluate if they would restore mitochondrial metabolism: On day 21, TMRM signal was increased (*P* < 0.0001; Fig. 1a) and ROS production was decreased (*P* < 0.0001; Fig. 1b and P < 0.0001; Fig. 1c) in the MSC + M0 + cPE group when compared to MSC + cPE. These results suggest a positive effect of macrophages on mitochondrial metabolism following exposure to cPE. The flow cytometry data confirmed the results of ROS production (Fig. 1c)—except for the MSCs groups at day 7.

### Macrophages promoted glycolytic reprogramming after exposure to cPE

**Fig. 2:**
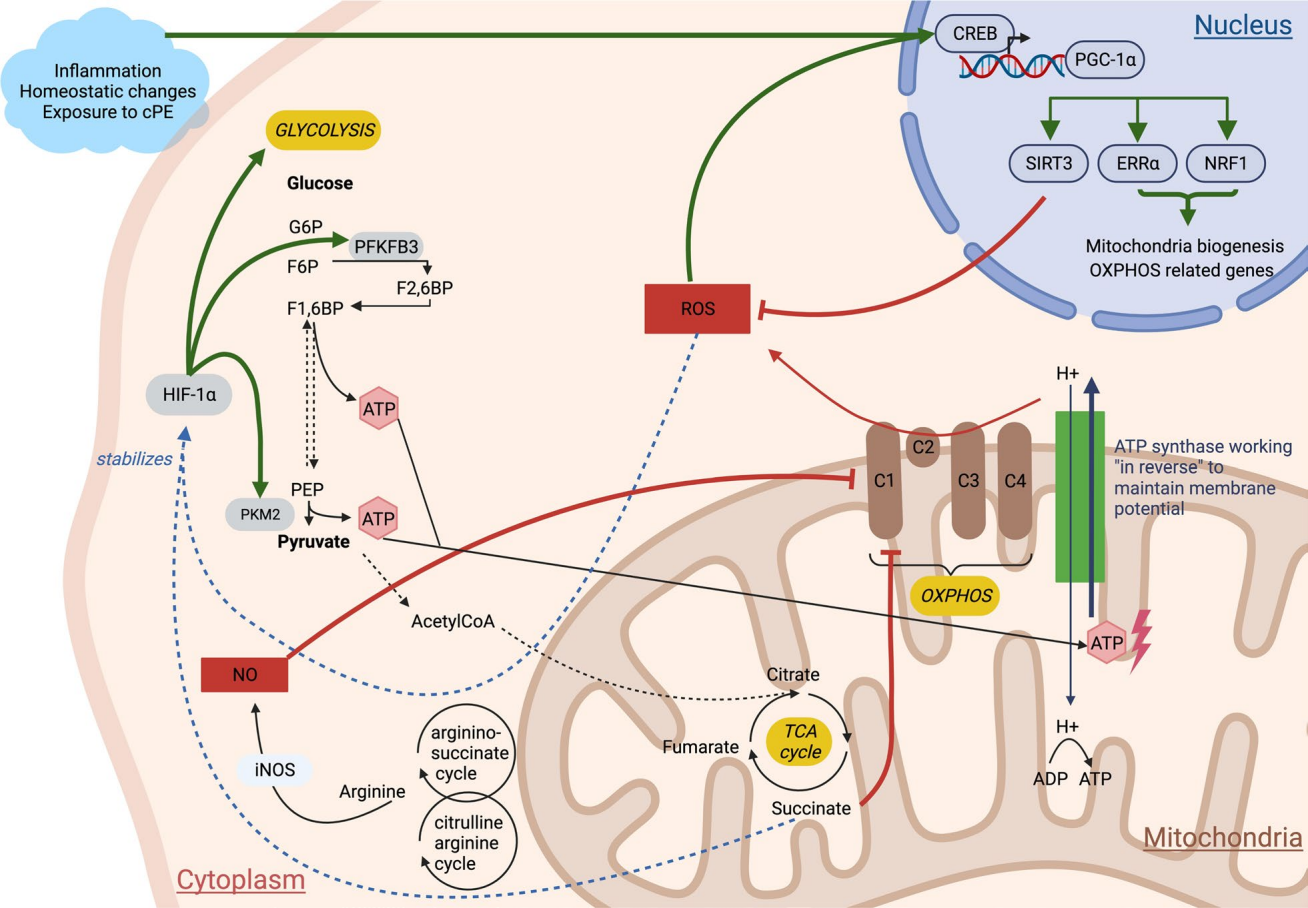
Metabolic changes following inflammatory stimulus. Glycolytic pathway is increased by HIF-1a, PKM2 and PFKFB3. OXPHOS is reduced. TCA metabolites accumulate: succinate promotes ROS production via inhibiting the OXPHOS pathway and stabilizes HIF-1a. Other cycles (arginine–succinate and citrulline–arginine) generate TCA metabolites but also produce arginine that activates iNOS expression and NO synthesis. NO inhibits OXPHOS, thus increasing ROS production. ATP from glycolysis is used by the ATP synthase to maintain membrane potential: ATP synthase works “in reverse.” ROS and homeostatic changes activate CREB transcription that activates PGC-1a expression. PGC-1a forms functional complexes with SIRT3, ERRα and NRF1 to reduce ROS production, enhance mitochondria biogenesis and restore OXPHOS. Figure created with BioRender.com. ATP: Adenosine 5'-Triphosphate, CREB: cAMP-Response Element-Binding Protein, ERRα: Estrogen-Related Receptor alpha, HIF-1α: Hypoxia-Inducible Factor 1-alpha, iNOS: Inducible Nitric Oxide Synthase, NO: Nitric Oxide, NRF1: Nuclear Respiratory Factor 1, OXPHOS: Oxidative Phosphorylation, PFKFB3: 6-Phosphofructo-2-Kinase/Fructose-2,6-Biphosphatase 3, PGC-1α: Peroxisome Proliferator-Activated Receptor-γ Coactivator, PKM2: Pyruvate Kinase M2, ROS: Reactive Oxygen Species, TCA: Tricarboxylic Cycle

**Fig. 3:**
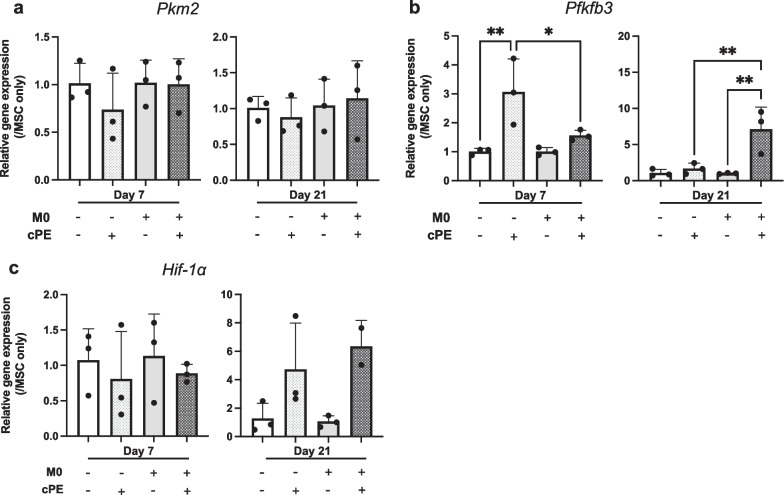
Metabolic switch to glycolytic pathway in MSCs as revealed by RT-qPCR after exposure to M0 macrophages and/or cPE, **a**: Pkm2; **b**: Pfkfb3; and **c**: Hif-1α, Values are means ± SD (n = 3). *p < 0.05; **p < 0.01. One-way ANOVA followed by post hoc Turkey test between MSC and MSC + cPE; MSC + M0 and MSC + M0 + cPE; and MSC + cPE and MSC + M0 + cPE. HIF-1α: Hypoxia-Inducible Factor 1-alpha, M0: naïve macrophages, cPE: contaminated polyethylene particles, PFKFB3: 6-Phosphofructo-2-Kinase/Fructose-2,6-Biphosphatase 3, PKM2: Pyruvate Kinase M2

The Warburg effect—also called aerobic glycolysis—is described as the increase of the glycolytic pathway under normal oxygen concentration. When exposed to inflammatory stimuli, cells increase their metabolic needs and switch their baseline oxidative phosphorylation to glycolysis: This allows a faster, less energy consuming but less efficient generation of ATP (Fig. 2). When undergoing osteogenic differentiation, MSCs require higher ATP levels [26]; thus, we analyzed the expression of glycolytic related genes with RT-qPCR to determine if exposure to cPE promoted the Warburg effect in order to provide enough energy for osteogenesis. PKM2 is a key enzyme of glycolysis; it catalyzes the last irreversible step of glycolysis that produces one molecule of ATP and one molecule of pyruvate. PKM2 is a rate-limiting enzyme; its concentration affects the overall rate of all the glycolytic pathway. We did not find differences in *Pkm2* expression among groups or among timepoints (Fig. 3a), suggesting that the presumed increased need in ATP production was not significant enough to impact PKM2 expression. PFKFB3 is a coenzyme to PFK1 that catalyzes the second step of glycolysis transforming F6P to F16BP. We found a significant increase in *Pfkfb3* expression following cPE exposure (Fig. 3b), suggesting an increase in the glycolytic pathway. HIF-1α is key transcription factor that regulates the balance between OXPHOS and glycolysis, even under normal oxygen concentration [27]. Although non-significant (Fig. 3c, ns), we found a trend in increased *Hif-1α* expression following cPE exposure. At day 21, gene expression levels for *Pkm2* (ns), *Pfkfb3* (*P* < 0.01*)* and *Hif-1α* (ns) were higher in the MSC + M0 + cPE group compared to the MSC + cPE group. Overall, these results suggest a glycolytic reprogramming when MSCs are exposed to cPE, and that macrophages enhance this phenomenon.

### Macrophages modulated mitochondrial biogenesis after exposure to cPE

**Fig. 4:**
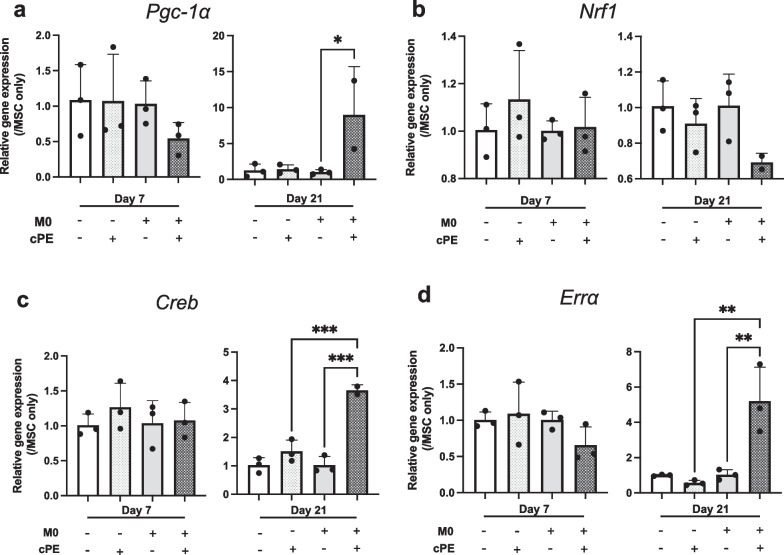
Changes in MSC mitochondrial metabolism as revealed by RT-qPCR after exposure to M0 macrophages and/or cPE. Changes in gene expression of **a**: Pgc-1α; **b**: Nrf1; **c**: Creb; and **d**: Errα. Values are means ± SD (n = 3). *p < 0.05; **p < 0.01. ***p < 0.001. One-way ANOVA followed by post hoc Turkey test between MSC and MSC + cPE; MSC + M0 and MSC + M0 + cPE; and MSC + cPE and MSC + M0 + cPE. CREB: cAMP-Response Element-Binding Protein, ERRα: Estrogen-Related Receptor alpha, M0: naïve macrophages, NRF1: Nuclear Respiratory Factor 1, cPE: contaminated polyethylene particles, PGC-1α: Peroxisome Proliferator-Activated Receptor-γ Coactivator

We analyzed several genes that not only regulate mitochondrial biogenesis and the oxidative phosphorylation pathway but function as nuclear transcription factors, thus regulating cellular processes such as cytokine signaling (Fig. 2). *Pgc-1α* is a master regulator of mitochondrial biogenesis, and its expression was significantly increased in the MSC + M0 + cPE group at day 21 (Fig. 4a), suggesting a positive effect of macrophages on mitochondrial metabolism—consistent with the mitochondrial staining data. PGC-1α also interacts with several transcription factors, including NRF1, ERRα and CREB. When exposed to cPE, we did not find significant modification in *Nrf1* expression, suggesting that the PGC-1α/NRF1 pathway was not involved in our model (Fig. 4b). But we found a significant increase of *Creb* (Fig. 4c) and *Errα* (Fig. 4d) at day 21 in the MSC + M0 + cPE group, supporting our theory of an enhanced modulating response to stress mechanisms induced by macrophages in the presence of cPE. CREB is a key transcriptional factor regulating PGC-1α expression. There is a relationship between NRF1, CREB and PGC-1α [28]: With exposure to an inflammatory stimulus, ROS production is increased and enhances CREB transcription that subsequently activates PGC-1α and NRF1 expression. PGC-1α regulates ERRα transcription and the ERRα/PGC-1α complex promotes OXPHOS in tissues with high energy requirements. All these effectors: PGC-1α, ERRα, CREB, NRF1 not only regulate mitochondrial biogenesis but also help maintain redox homeostasis. Thus, their increase at day 21 in the MSC + M0 + cPE group may indicate an effect of macrophages on the induction of redox homeostasis factors to counteract the glycolytic reprogramming, to restore a more efficient pathway in energy production.

### Macrophages polarized to both M1 and M2 phenotypes

**Fig. 5:**
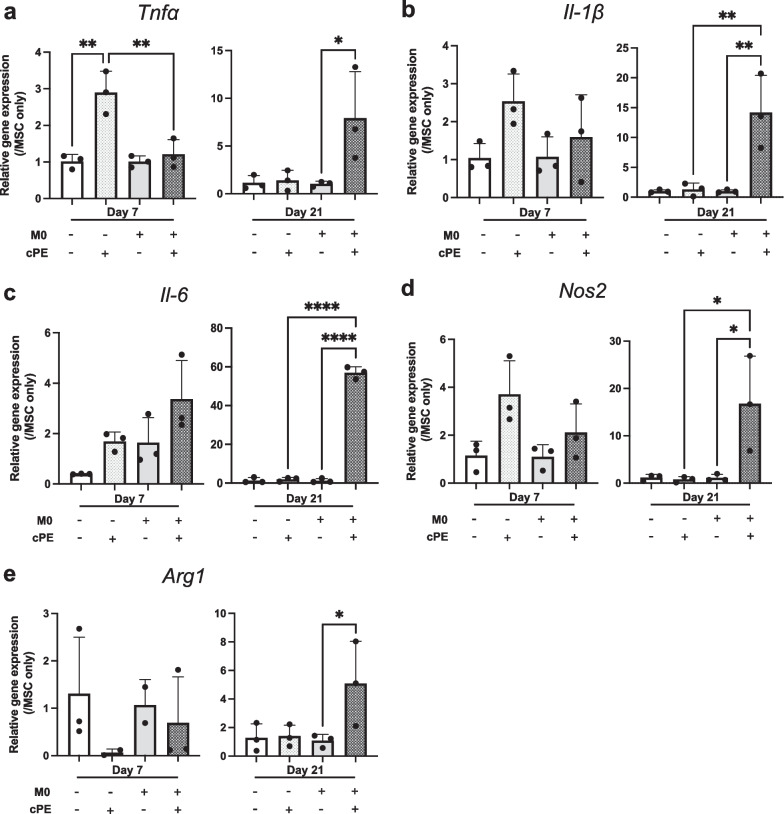
Macrophage polarization as revealed by RT-qPCR analysis after coculture and exposure to cPE. Changes in gene expression for pro-inflammatory markers: **a**: Tnfα; **b**: Il-1β; **c**: Il-6; **d**: Nos2 and anti-inflammatory markers; and **e**: Arg1. Values are means ± SD (n = 3). *p < 0.05; **p < 0.01; ****p < 0.0001. One-way ANOVA followed by post hoc Turkey test between MSC and MSC + cPE; MSC + M0 and MSC + M0 + cPE; and MSC + cPE and MSC + M0 + cPE. Arg1: Arginase 1, cPE: contaminated Polyethylene, IL-1β: Interleukin 1 beta, IL-6: Interleukin 6, MSCs: Mesenchymal Stromal Cells, Nos2: Nitric Oxide Synthase 2, TNFα: Tumor Necrosis Factor alpha, M0: naïve macrophages

Macrophages can polarize from an M0 uncommitted phenotype to a M1 pro-inflammatory or M2 anti-inflammatory phenotype. Each phenotype is defined by characteristic cytokine expression and histological markers. We added M0 macrophages in our experiments to analyze if the macrophages would polarize into M1 and/or M2, and how their phenotype would modulate (a) inflammation, (b) osteogenesis and (c) energy production pathways. TNFα is rapidly expressed following a pro-inflammatory stimulus: TNFα regulates macrophages activation and promotes their function by orchestrating the production of a pro-inflammatory cytokine cascade [29]. This is probably why only *Tnfα* expression was significantly increased following cPE exposure at day 7 (Fig. 1a), while other cytokines expression was not altered yet. But at day 21, the gene expression of M1 markers *Tnfα*, *Il-1β*, *Il-6* and *Nos2* all displayed a significant increase in the MSC + M0 + cPE group, suggesting an M1 polarization due to exposure to cPE (Fig. 5a–d). Interestingly, we also found a significant increase in the M2 marker *Arg1* at day 21 (Fig. 5e). These data suggest that in the coculture groups, macrophages mostly polarized to the M1 phenotype when exposed to cPE, but also expressed markers, to a lesser extent, of an M2 phenotype.

**Fig. 6:**
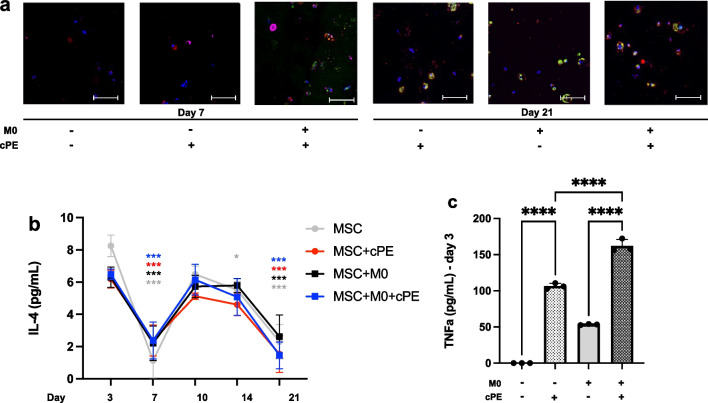
Macrophage polarization: immunohistochemical staining and cytokine secretion after exposure to M0 macrophages and/or cPE. **a**: Representative images of DAPI (blue, nucleus), CD11b (purple, macrophage), iNOS (red, M1) and Arg1 (green, M2) staining. Scale bar 50 µm. **b**: Changes in IL-4 cytokine secretion. **c**: Changes in TNFα cytokine secretion. Values below threshold from day 7. Values are means ± SD (n = 4 per condition). *p < 0.05; ****p < 0.0001. One-way ANOVA followed by post hoc Turkey test between all groups. DAPI: 4',6-Diamidino-2-Phenylindole, Dihydrochloride, IL-4: Interleukin 4, M0: naïve macrophages, cPE: contaminated polyethylene particles, TNFα: Tumor Necrosis Factor alpha

Immunohistochemical analysis revealed macrophages (DAPI^+^/CD11b^+^ cells) at day 7 in the coculture group, with both M1 (DAPI^+^/CD11b^+^/iNOS^+^) and M2 (DAPI^+^/CD11b^+^/Arg1^+^) (Fig. 6a). But no DAPI^+^/CD11b^+^ cells were found at day 21. Macrophages produce cytokines to regulate the local environment. Thus, we collected the supernatant to analyze cytokine secretion via ELISA. The anti-inflammatory cytokine IL-4 secretion peaked from day 10 to 14, before decreasing. Although we did not find a treatment effect, there was a significant time effect of IL-4 secretion when compared to baseline values at day 3 (Fig. 6b). The pro-inflammatory cytokine TNFα was only detectable at day 3, and its secretion was increased in both cPE groups, with a significant increase in the MSC + M0 groups (Fig. 6c).

### Macrophages did not affect the capacity for osteogenic differentiation of MSCs

**Fig. 7:**
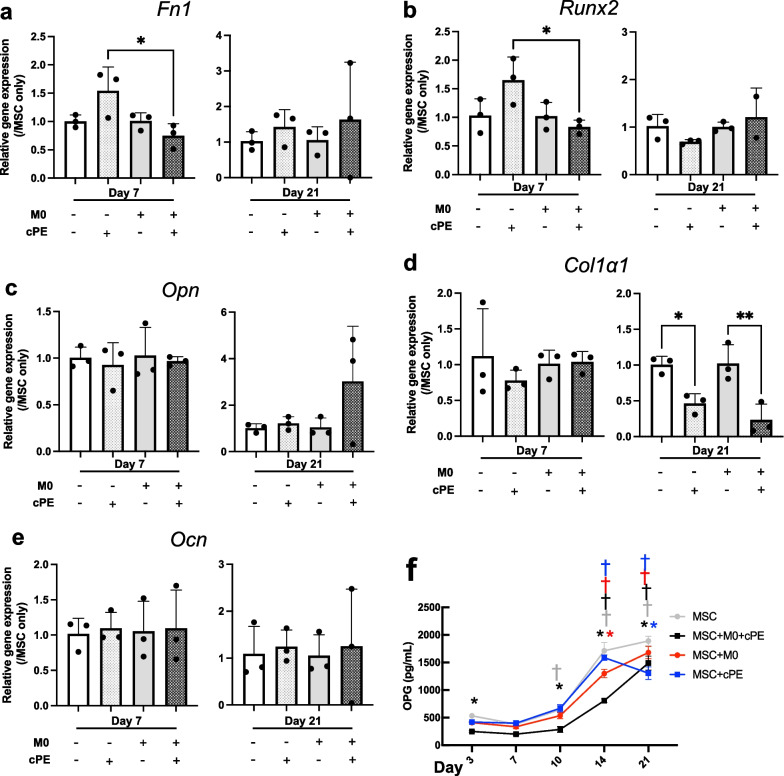
Osteogenic differentiation of MSCs as revealed by RT-qPCR analysis and cytokine secretion after exposure to M0 macrophages and/or cPE. Changes in expression of osteogenic differentiation-related genes. **a**: Fn1; **b**: Runx2; **c**: Opn; **d**: Col1a1; and **e**: Ocn. Values are means ± SD (n = 3). *p < 0.05; **p < 0.01. One-way ANOVA followed by post hoc Turkey test between MSC and MSC + cPE; MSC + M0 and MSC + M0 + cPE; and MSC + cPE and MSC + M0 + cPE. **f**: changes in OPG cytokine secretion. One-way ANOVA followed by post hoc Turkey test compared to values at day 3. Values above threshold after day 10. Collα1: Collagen 1, cPE: contaminated Polyethylene Particles, FN1: Fibronectin 1, M0: naïve macrophages, MSCs: Mesenchymal Stromal Cells, OCN: Osteocalcin, OPG: Osteoprotegerin/TNFRSF11B, OPN: Osteopontin, RUNX2: Runt-Related Transcription Factor 2

Among their differentiation capacities, we focused on osteogenic commitment of MSCs. Macrophages mitigate the cPE-induced chronic inflammatory osteolysis by enhancing osteogenic differentiation of MSCs. The qPCR analysis of RNA extracted from the 3D constructs demonstrated the expression of osteogenic markers. The expression of the uncommitted MSC marker *Fn1* and early markers of osteogenic differentiation *Runx2* and *Opn* did not display significant changes between groups and over time (Fig. 7a–c). Osteoblasts produce type I collagen, encoded by Collα1 gene. At day 7, *Collα1* gene expression was comparable between groups, but at day 21, *Collα1* expression was significantly decreased in both exposed to cPE groups (Fig. 7d). The late osteoblastogenesis marker *Ocn* did not show changes in expression among groups over time (Fig. 7e).

**Fig. 8:**
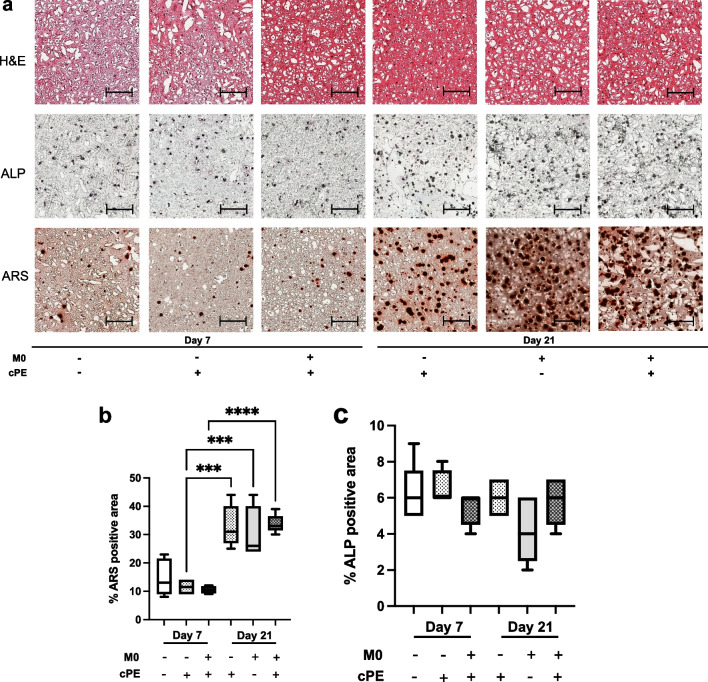
Osteogenic differentiation of MSCs by histological analysis. **a**: Representative images of H&E, ALP, and ARS staining. Scale bar 25 μm. **b**: Values are % of ARS-positive area at threshold 75. **c**: Values are % of ALP-positive area at threshold 75. Values are means ± SD (n = 4 per condition). *p < 0.05; ****p < 0.0001. One-way ANOVA followed by post hoc Turkey test between all groups. ALP: Alkaline Phosphatase, ARS: Alizarin Red, cPE: contaminated Polyethylene Particles, H&E: hematoxylin and eosin, M0: naïve macrophages

The ELISA analysis of the secretion of TNFRSF11B = osteoprotegerin (OPG), another marker of osteoblastic lineage, revealed a significantly increased secretion over time, suggesting a commitment to osteogenic differentiation among groups (Fig. 7f). At day 21, OPG secretion was comparable between MSC and MSC + M0 and between MSC + cPE and MSC + M0 + cPE and was significantly higher in MSC and MSC + M0 than in MSC + cPE and MSC + M0 + cPE. These data suggest that although the trend for increased OPG secretion was observed in all groups, cPE negatively affected OPG secretion and that addition of macrophages did not reverse this phenomenon. The H&E staining confirmed that cells were evenly distributed in the scaffolds. The ARS staining that evaluated tissue mineralization concurred with these results because they showed a significant increase in ARS-positive area for all analyzed groups at day 21 compared to day 7 (Fig. 8a and b). But the ALP staining, used to assess osteogenic differentiation, did not reveal any significant change (Fig. 8a and c).

## Discussion

We used validated GelMA parameters [30–32] and a previously described 3D model [7, 8] and found that: (a) mitochondrial metabolism was initially impaired when MCSs were exposed to cPE and (b) macrophages positively affected mitochondrial functional integrity and limited ROS production at a later time. Our results suggest that cells underwent a metabolic switch to glycolysis in the presence of macrophages and cPE. A potential explanation for the steady expression of the rate-limiting enzyme PKM2 could be that the change in glycolytic flux—suggested by PFKFB3 and HIF-1α—did not reach PKM2 functioning upper limit. But our model also triggered compensatory mechanisms: Response to stress genes expression was increased, suggesting a stimulated mitochondrial biogenesis and an attempt to restore OXPHOS signaling pathways.

Under inflammatory conditions, macrophages change their bioenergetic pathways: M1 macrophages reprogram their metabolism to aerobic glycolysis and reduce oxidative phosphorylation (i.e., mitochondrial respiration) [15, 19, 33]. First, increased expression of HIF-1α and PKM2 enhances glycolysis [15, 34]. Second, reduced OXPHOS rate leads to an accumulation of TCA metabolites: Succinate promotes ROS production and HIF-1α stabilization [35]. Third, the arginine–succinate and citrulline–arginine cycles produce fumarate (an intermediate of the TCA cycle) but also arginine that activates iNOS expression and NO synthesis [33]. NO perpetuates the phenomenon by inhibiting the respiratory chain, increasing ROS production and succinate accumulation. Lastly, at the level of the mitochondrial electron transport chain, the entire pathway starts working “in reverse”: ATP produced from glycolysis is transformed to ADP by the ATP synthase to maintain mitochondrial membrane potential, and the disruption of electron transfer across mitochondrial complexes balance leads to superoxide generation and subsequent mitochondrial ROS production.

A recent study confirmed that macrophage mitochondrial function was altered in an inflammatory environment [36]: Mitochondrial membrane potential was decreased in macrophages after LPS treatment. The authors suggested that TCA intermediate metabolites enhanced ROS production, which inhibited OXPHOS and dysregulated the mitochondrial energy metabolism, leading to a drop of membrane potential. Our day 7 result—increased ROS production and decreased membrane potential—is consistent with these studies.

Macrophages also polarize to an M2 anti-inflammatory phenotype to mitigate inflammation. At a metabolic scale, M2 macrophages have an intact TCA that provides substrates for oxidative phosphorylation and ATP production. Also known as the “master regulator of mitochondrial biogenesis,” PGC-1α is a connecting hub between mitochondrial biogenesis and metabolic pathways [37]. ROS and enhanced oxidative stress environment activate CREB transcription that induces PGC-1α expression [28, 37]. And PGC-1α creates “functional complexes” with ERRα and NRF1 to promote mitochondrial biogenesis, activity and OXPHOS [28, 38–40]. These responses to stress transcription factors are activated by inflammatory stimuli and help cells mitigate the metabolic vicious circle.

We found that osteogenic differentiation and bone formation were comparable with addition of different macrophage phenotypes. In previous studies, adding M0, M1 or M2 macrophages promoted osteogenic differentiation of MSCs after 4 weeks in a 3D model; ARS and micro-CT analysis revealed significant increases in all coculture groups compared to MSCs alone, and pro-inflammatory M1 macrophages enhanced bone formation most effectively [8]. Another 3D model revealed that adding cPE reduced bone mineralization of MSCs and expression of osteogenic markers OCN and RUNX2, and that, among all polarization phenotypes, M2 macrophages had the strongest effect on bone formation, assessed by micro-CT, and on osteogenic differentiation, increased expression of OPN and RUNX2 [7]. They reported a significant increase in OPN expression in MSC + M0 + cPE group after 4 weeks; our results show the same trend at day 21. Collα1 is a bone matrix protein, and its expression increases from uncommitted MSCs to pre-osteoblasts and to osteoblasts [41]. We found that Collα1 transcription was significantly decreased in the cPE groups at day 21. Our results are consistent with the above-mentioned studies: Uncommitted macrophages have a positive effect on MSCs osteogenic differentiation, and cPE exposure mitigates this cross talk.

Uncommitted macrophages can polarize in a 3D in vitro environment [8]. Our results suggest that M0 macrophages polarized to both M1 and M2 phenotypes; gene expression of M1 and M2 markers was increased, and cytokine secretion was measured. The changes in mitochondrial metabolic pathways discussed above are consistent with this hypothesis [15]. Inflammatory cytokine TNFα was observed only at day 3. Serum levels of TNFα dramatically increased and peaked shortly (1 h) after LPS stimulation, but rapidly decreased in a murine model [42]. Donaldson et al. [43] studied the immunomodulatory properties of GelMA by comparing TNFα secretion by macrophages in a 2D and in a 3D GelMA system. Following LPS stimulation, TNFα secretion was significantly lower in the 3D setup. A cell-free experiment in which the authors added TNFα to the culture medium revealed that TNFα concentration was decreased in the GelMA group. They explained this “mop up” effect by the fact that TNFα binds with GelMA, thus decreasing its concentration in the culture medium. TNFα is an early pro-inflammatory cytokine whose release is affected by GelMA; this “mop up” phenomenon is probably why we could not detect TNFα after day 3, although gene expression was still increased.

This study has limitations. We assessed the overall changes in mitochondrial metabolism in the culture systems without analyzing each cell type. MSC differentiation affects energetic metabolism; MSCs basal metabolism relies on glycolysis while osteogenic differentiation requires increased OXPHOS [44, 45]. Macrophage polarization modulates the balance between OXPHOS (M2 polarization) and glycolysis (M1 polarization) [15]. An inflammatory environment modifies cell metabolic fate [11]. MSC-macrophage cross talk and cPE exposure affect mitochondrial metabolism, and further analysis such as single-cell RNA sequencing may help in further understanding the signaling pathways of each cell type, the timeframe of those metabolic changes and how they influence each other. Also, only 2 scaffolds were used for the live cell mitochondrial staining. However, the fluorescent data captured using the confocal microscope can be considered reliable based on the power calculation, the amount of fluorescent data obtained from the confocal imaging acquisition and the 100 μm thickness of the z-stack analyzed. Additionally, we provided only indirect elements of macrophage polarization at day 21: gene expression of M1/M2 markers, metabolic changes and cytokine secretion. The in situ immunohistochemical markers could not be clearly detected at day 21. One most likely explanation is that macrophages underwent apoptosis when acute inflammation secondary to cPE exposure was resolved [46]. Another possibility is that the surface markers used for macrophage identification (CD11b, F4/80) could be involved in macrophage binding with the GelMA network. Finally, cell retrieval from GelMA can be challenging for flow cytometry based on our results, and even though we used validated protocols, cells died during the recovery process [47]. This might be because the bone formation that occurred within the scaffold changed its biomechanical properties and impaired successful cell retrieval.

This works raises several questions. Since macrophage polarization affects the osteogenic differentiation of MSCs in vitro [7], we can assume that already polarized macrophages would have contrasting influences on mitochondrial metabolism. Several studies revealed that immunomodulatory properties of MSCs could come from mitochondrial transfer [44, 48–50]; mitochondrial tracking could provide insight into the mechanism of MSC-macrophage cross talk. Also, the improvement of technologies to the field of 3D culture may facilitate the performance of glycolytic and ATP rate assays, thus improving our understanding of the balance between glycolysis and OXPHOS following cPE exposure and their relative contribution to ATP production.

## Conclusion

We found that cPE exposure alters mitochondrial metabolism and results in glycolytic reprogramming in a 3D coculture model of MSCs and macrophages. Macrophages cocultured with MSCs undergo metabolic changes to correct redox imbalances and restore homeostatic metabolism. This work revealed that the adverse effects of prolonged inflammation by an adverse stimulus (contaminated particles) on bone are due to dysregulated mitochondrial homeostasis, dysfunctional mitochondrial bioenergetics and metabolic reprogramming of macrophages. Novel strategies to modulate inflammation and mitochondrial bioenergetics for optimizing bone homeostasis in the presence of chronic inflammatory conditions could help mitigate these undesirable effects on bone.

## Data Availability

The datasets used and/or analyzed during the current study are available from the corresponding author on reasonable request.

## Abbreviations

3D: Three-dimensional
ADP: Adenosine diphosphate
ALP: Alkaline phosphatase
Arg1: Arginase 1
ARS: Alizarin Red
ATP: Adenosine 5′-triphosphate
BSA: Bovine serum albumin
Collα1: Collagen 1
cPE: Contaminated polyethylene particles = polyethylene particles coated with LPS
CREB: CAMP-response element-binding protein
DAPI: 4′,6-Diamidino-2-phenylindole, dihydrochloride
DI water: Deionized water
ERRα: Estrogen-related receptor alpha
FBS: Fetal bovine serum
FN1: Fibronectin 1
GelMA: Gelatin methacrylate
H&E: Hematoxylin and eosin
HIF-1α: Hypoxia-inducible factor 1-alpha
IL-1β: Interleukin 1 beta
IL-4: Interleukin 4
IL-6: Interleukin 6
iNOS: Inducible nitric oxide synthase
LPS: Lipopolysaccharides
MSCs: Mesenchymal stromal cells
NO: Nitric oxide
Nos2: Nitric oxide synthase 2
NRF1: Nuclear respiratory factor 1
OCN: Osteocalcin
OCT compound: Optimal cutting temperature compound
OPG: Osteoprotegerin/TNFRSF11B
OPN: Osteopontin
OXPHOS: Oxidative phosphorylation = mitochondrial respiration
PFK1: Phosphofructokinase-1
PFKFB3: 6-Phosphofructo-2-kinase/fructose-2,6-biphosphatase 3
PGC-1α: Peroxisome proliferator-activated receptor-γ coactivator
PKM2: Pyruvate kinase M2
ROS: Reactive oxygen species
RUNX2: Runt-related transcription factor 2
TCA: Tricarboxylic cycle = Krebs cycle = citric acid cycle
TMRM: Tetramethylrhodamine, methyl ester
TNFα: Tumor necrosis factor alpha
